# Dopaminergic Control of Locomotor Patterning during Development: A Tail for the Ages

**DOI:** 10.3389/fncel.2016.00095

**Published:** 2016-04-19

**Authors:** Aaron M. Lambert

**Affiliations:** ^1^Masino Lab, Neuroscience, University of Minnesota Twin CitiesMinneapolis, MN, USA; ^2^Engert Lab, Molecular and Cellular Biology, Harvard UniversityCambridge, MA, USA

**Keywords:** dopamine, locomotion, motor control, spinal cord, zebrafish, D4 receptor, development, developmental biology

Dopamine (DA) directly modulates motor circuits in the brain and spinal cord. While the somata of all dopaminergic (DAergic) neurons in zebrafish and mammals are supraspinal, some of these, termed diencephalospinal neurons (DDNs), send long-distance descending projections into the spinal cord (Figure 1A) and have recently been implicated in spinal network (Reimer et al., 2013) and locomotor development (Lambert et al., 2012) in zebrafish. After many years of seminal research on the morphology and genetic specification of zebrafish DDNs and the DAergic diencephalospinal tract (DDT) (Schweitzer and Driever, 2009), our recent study was the first to explicitly investigate DDN function (Lambert et al., 2012). We combined pharmacological DAergic perturbations, demarcated transections, and selective chemogenetic ablation of *orthopedia* (*otp*) neurons to demonstrate that conserved DAergic *otp* neurons–a subset of which are DDNs (Fujimoto et al., 2011; Figure 1A2)—provide the impetus for endogenous DA receptor 4 (D4R) signaling to initiate and maintain a developmental switch, between 80 and 96 h post fertilization (hpf), to the mature episodic locomotor pattern of zebrafish. Interestingly, Reimer et al. (2013) also demonstrated that DAergic *otp* neurons drive endogenous D4R signaling, confirmed both pharmacologically and genetically, but for an even earlier developmental function- to influence the differentiation of spinal motor progenitor cells between 24 and 48 hpf. Both studies used additional experimental approaches to demonstrate that D4R signaling directly in the spinal cord was sufficient to emulate the developmental processes under question: localized spinal application of DA or D4R agonists was shown to influence progenitor pools, when applied for many hours (Reimer et al., 2013), or to modulate the duration of locomotor episodes, when applied for just 5–10 min (Lambert et al., 2012). Collectively, these studies unveil that DDNs may drive endogenous spinal D4R signaling, via the DDT, for two temporally distinct roles in locomotor development: (1) a transient early role that acts directly on progenitor pools but not neurons, with long lasting consequences (Figure 1B, left), and (2) a late role that likely acts through ongoing spinal neuronal signaling, for maintenance of episodic locomotor patterns (Figure 1B, right). This commentary considers the extent to which an understanding of each of these developmental roles integrates with or is modified by the most recent DDN-related findings of Jay et al.

**Figure 1:**
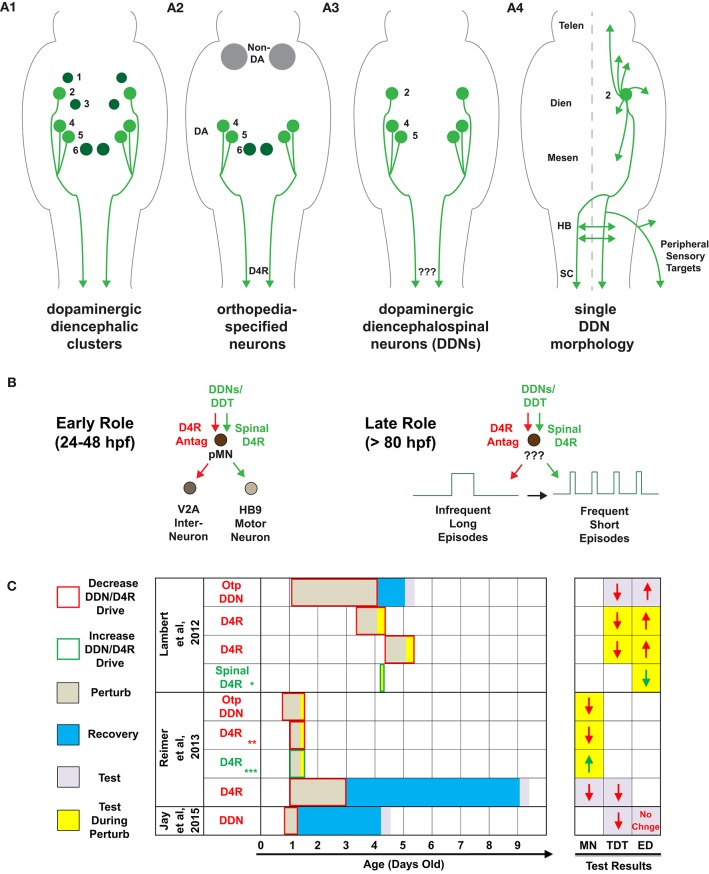
**Descending dopaminergic control of spinal network and locomotor development. (A)** Schematics of dorsal views of the zebrafish brain and spinal cord, by 4-days-old, of: **(A1)** dopaminergic diencephalic clusters (DC) DC1-DC6. Note that DC2, DC4, and DC5 are the exclusive dopaminergic diencephalospinal neurons (DDNs) that comprise the dopaminergic diencephalospinal tract (DDT). **(A2)** orthopedia-specified neurons in the Tg(otpb.A:nfsB–egfp)^*zc*77^ line used in Lambert et al. (2012), combined with systemic and localized dopamine receptor (DAR) pharmacology. **(A3)** DDNs targeted in Jay et al. (2015), but with no DAR pharmacology. **(A4)** single cell morphology of DC2 DDNs that tile virtually the entire rostrocaudal central neuraxis, as well as the peripheral sensory targets of the otic capsule, lateral line, and head and trunk neuromasts. **(B)** Putative model of the role of DDN-spinal D4R signaling in: (**B**, left) spinal neuron differentiation (24–48 hpf) via direct influence on spinal motor progenitor cells (pMNS) and (**B**, right) locomotor development (80–96 hpf) via influence on unknown spinal targets. **(C)** Timeline of DDN/D4R perturbations, recovery, and testing for the studies specified. Test results denote changes, at the color-coded time of testing, in the number of HB9 motor neurons (MN), total distance traveled (TDT), and episode duration (ED) as a function of each DDN/D4R perturbation. The Otp DDN perturbation in Lambert et al. (2012) was via chemogenetic ablation in the Tg(otpb.A:nfsB–egfp)^*zc*77^ line, whereas in Reimer et al. (2013) was via otpa^*m*866^ mutants (where otpa expression begins at 18 hpf). All perturbations labeled as D4R were systemic pharmacological D4R antagonism/agonism, except: 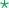 localized application of D4R agonist to transected spinal cord, 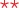 and 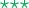 in addition to systemic pharmacology, recapitulated via D4Ra mRNA knockdown and rescue (respectively), 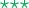 recapitulated via localized application of D4R agonist to transected spinal cord in an adult zebrafish model of spinal cord injury. Abbreviations in **(A4)** denote the following: telencephalon (Telen), diencephalon (Dien), mesencephalon (Mesen), hindbrain (HB), and spinal cord (SC).

Since the later role of DDNs suggests a mechanism of ongoing DAergic neurotransmission to sculpt episodic locomotion, monitoring activity patterns of DDNs during neural locomotor output *in vivo* could elucidate potential DDN-spinal locomotor circuit dynamics. The recent study by Jay et al. was the first to do this in any animal model. However, activity patterns of murine DAergic mesostriatal neurons (DMNs) have been studied extensively and show that they exhibit two distinct outputs: phasic bursting that acts on low-affinity D1-like receptors, and tonic firing that acts on high-affinity D2-like receptors (Dreyer et al., 2010). Jay et al. found that zebrafish DDNs also exhibit phasic and tonic outputs, which plausibly may also differentially drive D1-like and D2-like signaling, respectively. Since, modulation of locomotor episode durations in zebrafish requires D2-like D4R signaling (Lambert et al., 2012), one would hypothesize DDN tonic firing to drive this phenomenon. Interestingly, periods of fictive locomotor activity correlated to DDN phasic bursting, but not tonic firing (Jay et al., 2015). However, further examination revealed that DDN phasic bursting did not reliably drive locomotor episodes or modulate their durations. Moreover, DDN tonic firing and locomotor inactivity each occurred the overwhelming majority of the time, so any correlation between the two is likely misleading. Instead, DDN tonic firing may modulate motor output—on a slower timescale—by setting spinal neuromodulatory tone (Hauber, 2010), rather than canonical time-locked millisecond precision to motor circuits. Taken together, if DDN activity directly influences the number and/or the duration of locomotor episodes, it likely does so via its prevalent tonic firing that is perhaps partially driven cell-autonomously.

Next, Jay et al. assessed the behavioral consequences of laser ablating DDNs (Figures 1A3,A4). We had previously chemogenetically ablated *otp* neurons—some of which are DDNs (Figure 1A2)—and assessed behavioral and neural locomotor output (Lambert et al., 2012). Both studies found that ablation of DDNs depressed locomotor output, but only our study found an increase in the duration of individual locomotor episodes (Figure 1C). This discrepancy could be due to: (1) differences in timing of ablations and assessments, (2) ablation of non-DDNs in our study, and/or (3) differences in behavioral acquisition and analyses. Our study assessed behavior at 1 day post-ablation (dpa), at 5-days-old, whereas Jay et al. assessed behavior at 3 dpa, at 4-days-old. The latter may be confounded by homeostatic compensation; a recent study showed that widespread chemogenetic ablation of DAergic neurons altered multiple locomotor parameters at 1 dpa, some of which recovered by 3 dpa and coincided with newborn DAergic neurons (Godoy et al., 2015). Furthermore, 3 dpa of DDNs could allow for developing DAergic projections to the hindbrain to homeostatically extend into the spinal cord, or induce a shift from supraspinal (Kimura et al., 2013) and spinal (Lambert et al., 2012) modulation of episode duration, to more exclusive supraspinal control. Alternatively, it is possible that non-DDN DAergic *otp* neurons, which Jay et al. did not target, act supraspinally for the *in vivo* DAergic modulation of spontaneous episode durations that we and others have reported (Thirumalai and Cline, 2008; Lambert et al., 2012; Lange et al., 2012; Decker et al., 2014), including previous work from the same lab as in Jay et al. (Tong and McDearmid, 2012). While, Jay et al. did not explore any DA receptor (DAR) or localized spinal mechanisms, our study recapitulated the consquences of *otp* ablations on episode duration via systemic and localized DAR pharmacology to the transected spinal cord (Lambert et al., 2012); these manipulations also circumvented any caveats of recovery in the DDN or *otp* ablation experiments (Figure 1C). Aside from any of the above, the discrepancy of behavioral results between studies could simply be due to our study recording multiple fish in a 50 mm arena, compared to Jay et al. recording fish in isolation in a five-fold smaller arena. Each study also employed different definitions and detections of free-swimming episode durations. Moreover, higher acquisition rates increase fidelity of episodic structure, and Jay et al. only acquired videos at 15 Hz whereas we acquired at 60 Hz. Additionally, we also confirmed altered fictive episode durations acquired at 10,000 Hz.

Independent of any role of DDNs in episode duration, it is not clear whether Jay et al.'s result of depressed locomotor output at 3 dpa of DDNs is linked to the loss of ongoing DDN neuronal activity at 4-days-old, or is simply a long-term consequence of perturbing the early influence of DDNs on progenitor pools from 24 to 48 hpf (Figure 1C). Reimer et al. (2013) showed that transiently blocking endogenous D4R signaling from 24 to 72 hpf resulted in depressed locomotor output 6 days later that coincided with altered spinal neuronal distributions (Figure 1C). Hence, future studies interrogating the role of ongoing DDN neuronal activity on locomotor output should ablate or optogenetically manipulate DDNs only after the critical period for early DDN influence of progenitor pools has passed.

Finally, an inherent caveat of investigating the function of vertebrate DDNs is that their conserved morphology tiles most of the rostrocaudal extent of the central nervous system, from telencephalon to spinal cord (Takada et al., 1988; Tay et al., 2011; Figure 1A4). As such, it is plausible that DDNs simultaneously modulate spatially disparate postsynaptic targets, both spinally and supraspinally. Additionally, Jay et al. elegantly revealed that DDNs also likely innervate peripheral sensory structures (Figure 1A4). Hence, future studies testing whether DDNs directly modulate spinal locomotor circuits *in vivo* should circumvent any potential DDN influences on supraspinal or peripheral DAergic signaling. This could be achieved via laser ablation or optogenetic manipulation of DDT axons specifically at the level of the spinal cord. It will be then that sources of spinal DA can finally speak unambiguously about their spinal locomotor influences.

## Author contributions

The author confirms being the sole contributor of this work and approved it for publication.

## Funding

This work was supported by my competitive predoctoral fellowship on the MnDRIVE Neuromodulation Initiative, as well as NINDS funding from Florian Engert (5U01NS090449-02), my postdoctoral advisor.

### Conflict of interest statement

The author declares that the research was conducted in the absence of any commercial or financial relationships that could be construed as a potential conflict of interest.

## References

[B1] Decker A. R. McNeill M. S. Lambert A. M. Overton J. D. Chen Y.-C. Lorca R. A.. (2014). Abnormal differentiation of dopaminergic neurons in zebrafish trpm7 mutant larvae impairs development of the motor pattern. Dev. Biol. 386, 428–439. 10.1016/j.ydbio.2013.11.015 24291744 PMC3971878

[B2] Dreyer J. K. Herrik K. F. Berg R. W. Hounsgaard J. D. (2010). Influence of phasic and tonic dopamine release on receptor activation. J. Neurosci. 30, 14273–14283. 10.1523/JNEUROSCI.1894-10.2010 20962248 PMC6634758

[B3] Fujimoto E. Stevenson T. J. Chien C.-B. Bonkowsky J. L. (2011). Identification of a dopaminergic enhancer indicates complexity in vertebrate dopamine neuron phenotype specification. Dev. Biol. 352, 393–404. 10.1016/j.ydbio.2011.01.023 21276790 PMC3069253

[B4] Godoy R. Noble S. Yoon K. Anisman H. Ekker M. (2015). Chemogenetic ablation of dopaminergic neurons leads to transient locomotor impairments in zebrafish larvae. J. Neurochem. 135, 249–260. 10.1111/jnc.13214 26118896

[B5] Hauber W. (2010). Dopamine release in the prefrontal cortex and striatum: temporal and behavioural aspects. Pharmacopsychiatry 43(Suppl. 1), S32–S41. 10.1055/s-0030-1248300 20480446

[B6] Jay M. De Faveri F. McDearmid J. R. (2015). Firing dynamics and modulatory actions of supraspinal dopaminergic neurons during zebrafish locomotor behavior. Curr. Biol. 25, 435–444. 10.1016/j.cub.2014.12.033 25639243 PMC4331284

[B7] Kimura Y. Satou C. Fujioka S. Shoji W. Umeda K. Ishizuka T.. (2013). Hindbrain V2a neurons in the excitation of spinal locomotor circuits during zebrafish swimming. Curr. Biol. 23, 843–849. 10.1016/j.cub.2013.03.066 23623549

[B8] Lambert A. M. Bonkowsky J. L. Masino M. A. (2012). The conserved dopaminergic diencephalospinal tract mediates vertebrate locomotor development in zebrafish larvae. J. Neurosci. 32, 13488–13500. 10.1523/JNEUROSCI.1638-12.2012 23015438 PMC3481997

[B9] Lange M. Norton W. Coolen M. Chaminade M. Merker S. Proft F.. (2012). The ADHD-susceptibility gene lphn3.1 modulates dopaminergic neuron formation and locomotor activity during zebrafish development. Mol. Psychiatry 17, 946–954. 10.1038/mp.2012.29 22508465

[B10] Reimer M. M. Norris A. Ohnmacht J. Patani R. Zhong Z. Dias T. B.. (2013). Dopamine from the brain promotes spinal motor neuron generation during development and adult regeneration. Dev. Cell 25, 478–491. 10.1016/j.devcel.2013.04.012 23707737

[B11] Schweitzer J. Driever W. (2009). Development of the dopamine systems in zebrafish, in Development and Engineering of Dopamine Neurons, Advances in Experimental Medicine and Biology, Vol. 651 (New York, NY: Springer), 1–14. 10.1007/978-1-4419-0322-8_1 19731546

[B12] Takada M. Li Z. K. Hattori T. (1988). Single thalamic dopaminergic neurons project to both the neocortex and spinal cord. Brain Res. 455, 346–352. 2900059 10.1016/0006-8993(88)90093-5

[B13] Tay T. L. Ronneberger O. Ryu S. Nitschke R. Driever W. (2011). Comprehensive catecholaminergic projectome analysis reveals single-neuron integration of zebrafish ascending and descending dopaminergic systems. Nat. Commun. 2:171. 10.1038/ncomms1171 21266970 PMC3105308

[B14] Thirumalai V. Cline H. T. (2008). Endogenous dopamine suppresses initiation of swimming in prefeeding zebrafish larvae. J. Neurophysiol. 100, 1635–1648. 10.1152/jn.90568.2008 18562547 PMC2544474

[B15] Tong H. McDearmid J. R. (2012). Pacemaker and plateau potentials shape output of a developing locomotor network. Curr. Biol. 22, 2285–2293. 10.1016/j.cub.2012.10.025 23142042 PMC3525839

